# Validation of Operational Definition to Identify Patients with Osteoporotic Hip Fractures in Administrative Claims Data

**DOI:** 10.3390/healthcare10091724

**Published:** 2022-09-08

**Authors:** Young-Kyun Lee, Jun-Il Yoo, Tae-Young Kim, Yong-Chan Ha, Kyung-Hoi Koo, Hangseok Choi, Seung-Mi Lee, Dong-Churl Suh

**Affiliations:** 1 Department of Orthopedic Surgery, Seoul National University Bundang Hospital, Seongnam 13620, Korea; 2 Department of Orthopedic Surgery, Gyeongsang National University Hospital, Jinju 52727, Korea; 3 Department of Orthopedic Surgery, Konkuk University Medical Center, Seoul 05030, Korea; 4 Department of Orthopedic Surgery, Chung-Ang University College of Medicine, Seoul 06974, Korea; 5 College of Pharmacy, Chung-Ang University, Seoul 06974, Korea; 6 College of Pharmacy, Daegu Catholic University, Gyeongsan 38430, Korea

**Keywords:** hip fractures, osteoporosis, validation, claims data, diagnosis codes

## Abstract

As incidences of osteoporotic hip fractures (OHFs) have increased, identifying OHFs has become important to establishing the medical guidelines for their management. This study was conducted to develop an operational definition to identify patients with OHFs using two diagnosis codes and eight procedure codes from health insurance claims data and to assess the operational definition’s validity through a chart review. The study extracted data on OHFs from 522 patients who underwent hip surgeries based on diagnosis codes. Orthopedic surgeons then reviewed these patients’ medical records and radiographs to identify those with true OHFs. The validities of nine different algorithms of operational definitions, developed using a combination of three levels of diagnosis codes and eight procedure codes, were assessed using various statistics. The developed operational definition showed an accuracy above 0.97 and an area under the receiver operating characteristic curve above 0.97, indicating excellent discriminative power. This study demonstrated that the operational definition that combines diagnosis and procedure codes shows a high validity in detecting OHFs and can be used as a valid tool to detect OHFs from big health claims data.

## 1. Introduction

Hip fractures, the second most common osteoporotic fractures after vertebral fractures, almost always require surgery [1]. As these fractures are associated with impaired mobility, increased mortality, and high medical expenses [2], they pose a considerable socioeconomic burden on the healthcare system in aging societies [3,4,5]. In 2000, there were an estimated 1.6 million osteoporotic hip fractures (OHFs) worldwide [6]. Following a global life expectancy increase, the incidence of hip fractures has increased and is projected to reach 4.5 million in 2050 [7]. This phenomenon is of particular concern in Asian countries [8,9,10,11].

Large-scale databases have been used for epidemiologic studies on OHFs [5,12,13,14]. In South Korea, several studies on the disease burden and treatment outcomes for osteoporosis and hip fractures have been conducted based on nationwide medical claims data [15,16,17,18,19]. Specifically, claims databases provide large-scale nationwide data without any selection or recall bias [20,21]. However, such data have potential limitations, including a lack of clinical information and coding errors, because the databases were established for reimbursements, not for medical research [18,22,23,24].

All South Korean citizens are eligible for coverage under the National Health Insurance Program (97%) or the Medical Aid Program (3%) [25]. A total of 53 million people are covered by those two programs and are included in the Health Insurance Review and Assessment (HIRA) database [21,26]. However, health insurance claims data cannot be used to differentiate osteoporotic and nonosteoporotic hip fractures because they do not include injury mechanisms, radiographs, or bone mineral density results [18,22,23,24]. To address this gap, researchers need to develop the appropriate algorithms to identify OHFs and provide evidence for the validity of their operational definition. Several studies have conceptually defined femoral neck and intertrochanteric fractures caused by falling from standing height as OHFs [27,28]. However, there is no standard operational definition for OHFs from previous studies, especially when using health insurance claims data on a large scale.

This study aimed to develop an algorithm to identify patients with OHFs among hip surgery patients using the International Classification of Diseases 10th edition (ICD-10) diagnosis codes and procedure codes for hip fracture surgeries of the health insurance claims data and assess this algorithm’s validity via chart reviews.

## 2. Materials and Methods

### 2.1. Data Source and Sample Patients

This study used the administrative claims data of inpatients and outpatients from Seoul National University Bundang Hospital, South Korea, an academic tertiary referral hospital. Patients aged 50 years or older who underwent hip surgeries between 1 January 2018 and 31 December 2018 were extracted from the administrative claims data. These patients were selected because OHFs are prevalent in this patient age group [3,15,29,30,31]. In addition, the dataset comprises demographic information, including age, sex, comorbidities described by diagnosis codes, and surgical procedures performed for treatment. This study was approved by the Institutional Review Board of Seoul National University Bundang Hospital (Approval Number: X-1801-447-90).

### 2.2. Diagnosis and Procedure Codes for the Definition of Osteoporotic Hip Fractures

This study follows the definition of osteoporotic fractures as femoral neck and intertrochanteric fractures caused by injuries from falling from equal to or less than standing height in patients at least 50 years old [27,28]. Both ICD-10 diagnoses and procedure codes were used to construct algorithms to identify OHFs, categorized by the codes for femoral neck fractures (S72.0) and intertrochanteric fractures (S72.1). Moreover, the algorithms used for identifying OHFs were developed based on algorithms used by previous studies and survey responses from an expert panel of 10 orthopedic surgeons [29,30,31,32,33,34].

Eight procedures for hip fracture surgeries were used to develop the operational definition for OHFs. Six surgical procedures for hip fractures were selected, including “open reduction of fractured extremity-femur (code N0601 for simple procedure and code N0611 for complex procedure)”, “closed pinning-femur (N0991)”, “external fixation-pelvis/femur (N0981)”, and “hemiarthroplasty-hip (N0715 for simple and N2710 for complex)” [18,23]. In addition, two procedures, including “total joint arthroplasty-hip (N0711 for simple and N2070 for complex)”, were also included due to the increasing number of total hip arthroplasties for hip fractures [13].

In the Korean National Health Insurance Service system, surgical procedure codes including “open reduction of fractured extremity-femur (complex)”, “hemiarthroplasty-hip (complex)”, and “total joint arthroplasty-hip (complex)”, were considered complex procedures when a patient had at least one complex condition. These complex conditions include patient conditions that increase the difficulty of surgery, such as chronic renal failure with prior or pending organ transplantation, cardiovascular stent with thrombolytic agent treatment, myocardial infarction/angina (Goldman cardiac risk Ⅲ or more), uncontrolled diabetes (HbA1C > 7.0), liver cirrhosis, blood cancer, hemophilia or coagulation abnormality, severe obstructive lung disease, history of venous thromboembolism, anticoagulant (higher level of aspirin) use due to cerebrovascular accident, rheumatoid arthritis under treatment (DAS28 > 5.1), peripheral arterial occlusive disease, progressive spinal cord paralysis or paralytic syndrome, pathologic fracture (primary, metastatic, or osteoporotic), infection sequela or periprosthetic joint infection, arthroplasty for bone defects measuring 1 inch or more, arthroplasty for bony deformities 15° or more, flexion contractures measuring 20° or more, revision after prior revision arthroplasty, and reoperation after the previous arthrodesis.

### 2.3. Algorithms to Identify Osteoporotic Hip Fractures

Nine algorithms using a combination of the ICD-10 diagnosis codes for femoral neck fractures or intertrochanteric fractures (S72.0 and S72.1) with eight procedure codes for hip fracture surgeries (N0601, N0611, N0991, N0981, N0715, N2710, N0711, and N2070) were developed to identify OHFs from administrative claims data. The levels of the diagnosis codes were defined as primary, secondary, or tertiary diagnostic codes according to the order recorded on the health insurance claim statement for each patient. The developed algorithms are as follows:

I. Algorithms using primary diagnosis codes.
  I-1: based on primary diagnosis codes only.
  I-2: based on primary diagnosis codes and eight procedure codes.
  I-3: based on primary diagnosis code or eight procedure codes.
II. Algorithms using primary or secondary diagnosis codes.
  II-1: based on primary or secondary diagnosis codes only.
  II-2: based on primary or secondary diagnosis codes and eight procedure codes.
  II-3: based on primary or secondary diagnosis codes or eight procedure codes.
III. Algorithms using primary, secondary, or tertiary diagnosis codes.
  III-1: based on primary, secondary, or tertiary diagnosis codes only.
  III-2: based on primary, secondary, or tertiary diagnosis codes and eight procedure codes.
  III-3: based on primary, secondary, or tertiary diagnosis codes or eight procedure codes.

Two orthopedic surgeons independently reviewed the enrolled patients’ medical records and imaging reports to confirm diagnosis consistency. The information reviewed included the sites and causes of the fractures, treatments, and imaging test results (radiography, bone scan, computerized tomography, resonance imaging, or bone densitometry). Femoral neck and intertrochanteric fractures caused by minor traumas, such as falls from standing height, were considered the gold standard to define OHFs [27].

### 2.4. Statistical Analysis

Patient characteristics were presented as frequencies and percentages or means and standard deviations (SDs) and were compared between males and females using a chi-square test and a *t*-test with a significance level of 0.05. The diagnostic evaluation algorithms’ sensitivity (true-positive rate) and specificity (true-negative rate) were calculated using a 2 × 2 table applied to the gold standard to evaluate their validity. Sensitivity is the proportion of true positive subjects with the disease among the total subjects with the disease, and it indicates a diagnostic test’s ability to recognize subjects with the disease. In addition, specificity is the proportion of subjects without the disease, identified through negative test results among the total subjects without the disease. It indicates the diagnostic accuracy of a test’s potential to recognize disease-free subjects [35].

The positive predictive value (PPV) and negative predictive value (NPV) were also calculated to compare the validity of each algorithm. The PPV, the probability that patients identified based on the OHF codes truly have OHFs, measures the precision of correctly identifying OHFs using claims data. It was calculated as the percentage of patients with OHFs confirmed by medical record reviews out of the total number of patients with OHFs identified using claims data [36]. Comparatively, NPV, the probability that patients without OHF codes truly do not have OHFs, was calculated as the proportion of patients without OHF confirmed by medical record reviews out of the total number of patients without hip fractures identified using claims data.

Accuracy, the global measure for correctly identifying patients, was calculated as the proportion of correctly identified patients (true positives and negatives in identifying hip fractures) among all study patients [36]. The overall performance of the operational definitions was also measured using the area under the receiver operating characteristic (AU-ROC) curve, which plots the tradeoff between sensitivity and specificity [37]. An area under the curve value of 1.0 corresponds to a perfectly accurate algorithm definition, whereas 0.5 corresponds to a random chance. Meanwhile, algorithms with an area under the curve between 0.9 and 1.0 have excellent discrimination abilities [35].

The 95% confidence interval (CI) was calculated based on the normal approximation of the binomial distribution [38]. All analyses were conducted using SAS statistical software (SAS Institute Inc., Cary, NC, USA).

## 3. Results

A total of 522 patients who underwent any form of hip surgery between January 2018 and December 2018 were included in the study. The patients’ mean age was 71.14 ± 11.74 years, and 65.9% were female (Table 1).

Female patients were significantly older than male patients, and hip fractures were diagnosed more frequently in female patients based on the primary, secondary, and tertiary diagnoses. The number of patients with hip fractures increased from 134 to 172 as the diagnosis code levels for identifying hip fractures increased from primary to tertiary diagnosis codes.

Table 2 shows 134 patients with hip fractures, 65 (12.5%) having femoral neck fractures (S72.0) and 69 (13.2%) with femoral intertrochanteric fractures (S72.1), as identified using primary diagnosis codes. Based on the primary, secondary, or tertiary diagnosis codes, 173 patients were identified with S72.0 (85 patients, 16.3%) or S72.1 (88 patients, 16.9%).

Table 3 shows the association between the number of patients with diagnosed hip fractures using the procedure codes and the number of patients with hip fractures confirmed by orthopedic surgeons. Among the 522 eligible patients, 134 were identified as patients with true OHFs after orthopedic surgeons reviewed their medical records and radiographs.

Table 4 provides the estimated sensitivity, specificity, PPV, NPV, accuracy, and AU-ROC curve for the different algorithms using different levels of diagnosis and procedure codes. In general, these estimates increase as higher levels of diagnosis codes (i.e., primary, secondary, or tertiary diagnosis codes) and procedure codes were used to identify patients with hip fractures. When the study used three levels of diagnosis codes (primary, secondary, and tertiary diagnosis codes) combined with procedure codes, the accuracy and AU-ROC curve value were >0.9 for all algorithms. Identification using diagnosis codes or procedure codes showed the lowest values in accuracy and AU-ROC curve in terms of all the different levels of the diagnosis codes.

**Figure 1:**
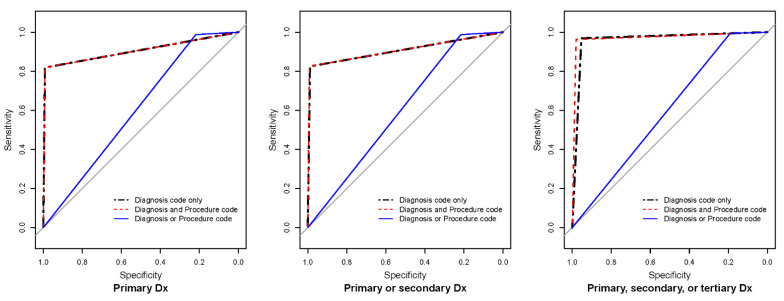
Receiver operator curves of the operational definition for osteoporotic hip fractures. Dx: diagnosis. Grey lines indicate chance diagonals (area under the curve = 0.5).

Figure 1 depicts the ROC curves of the nine developed algorithms using diagnosis and procedure codes. Regardless of diagnosis code level, the AU-ROC significantly differed between algorithms based on diagnosis only, diagnosis and procedure codes, and diagnosis or procedure codes.

## 4. Discussion

Identifying patients with OHFs using claims data is challenging because the established claims databases for reimbursements do not include a patient’s injury mechanism and bone mineral density test results. This study evaluated the validity of the algorithms for identifying patients with OHFs using national health claims data. Compared with the diagnosis codes alone, the combined operational definition of hip fracture surgery diagnosis and procedure codes showed superior validity results. In addition, the algorithm using primary, secondary, and tertiary diagnosis codes, and procedure codes showed better validity than using only the primary diagnosis codes. The definition of “diagnosis codes with procedure codes for hip fracture surgery” may be more appropriate for comparative studies evaluating the treatment efficacy among OHF patients because it had a higher validity score.

The PPVs of this study are comparable to the diagnoses (83.4%) of a previous validation study, which compared the accuracy between derived diagnoses based on the national claims database and patients’ medical records [24]. The PPVs of algorithms for identifying hip fractures using diagnosis codes have been reported as 20–96% for administrative claims data in other countries [39,40,41]. The complimentary use of hip fracture-related procedural codes improves the validity of case identification from the health insurance claims data [42]. In a study using US claims data for subtrochanteric femoral fractures, the positive predictive value of the algorithm, including surgeons’ diagnosis codes, was 15% higher than the algorithm including only the primary hospital discharge diagnoses [43]. However, the usefulness of procedure codes in detecting hip fractures may vary in different healthcare settings. In a study comparing methods of identifying hip fractures among nursing home patients using Medicare claims data, the addition of procedure codes resulted in a lower PPV than diagnosis codes alone [44]. This result may be attributed to avoiding fracture surgeries among some severe patients with end-stage conditions [45].

This study identified OHFs based on the S72.0 and S72.1 diagnosis codes. However, there is a possibility that other diagnosis codes were used despite the occurrence of OHFs. Nevertheless, identifying hip fractures using tertiary diagnosis codes showed that false negatives represent only 4.4% of hip fractures. Moreover, many previous observational studies have used the two codes to identify hip fractures from various administrative data [29,30,31,32,33,34,46]. Femoral neck and intertrochanteric fractures caused by low-energy traumas, such as falls from standing height, were used as the gold standard of OHFs by orthopedic surgeons involved in this study. Osteoporotic fractures are described as fractures that result from mechanical forces that normally do not cause fractures, known as low-level trauma, as mentioned in the National Institute for Health and Care Excellence (NICE) clinical guidelines [47]. The consideration of low-energy fractures as being osteoporotic is widespread, regardless of the patient’s bone mineral density, and bone densitometry may not be essential for diagnosing osteoporosis in these situations [27,48].

This study used both diagnosis and procedure codes in the algorithms. As the level of diagnosis codes increased, the validity of sensitivity, accuracy, and AU-ROC curve increased, but specificity and the PPVs did not increase. It was difficult to identify studies comparing the level of diagnosis codes (primary, secondary, etc.) to verify the validity of the hip fracture diagnoses. Nevertheless, in the latest study that evaluated the validity of the operational definition for distal radius fractures using the Korean National Health Insurance data, the sensitivity, specificity, and positive predictive value of the definition with all diagnosis codes were similar to those of the definition with primary and secondary codes [49]. Specifically, compared with the definition without procedure codes, the operational definition with all diagnosis and procedure codes showed lower sensitivity, higher specificity, and higher PPV. Among patients who underwent hip surgeries in this study, women were more likely to have diagnostic codes for hip fractures than men. This observation was presumed to result from a higher OHF incidence in women than in men, and this trend has been reported in epidemiologic studies in several countries [2,29,30,34]. This study involved patients aged 50 years or older to identify osteoporosis-related hip fractures because a previous study demonstrated that the incidence of hip fractures remarkably increased in Korean adults aged 50 and older [29].

The findings of this study should be interpreted with caution because of the limitations associated with the database. First, the database was from a single institute with a relatively small sample size, leading to low external validity. Although the chosen institute is a tertiary referral center, a substantial number of patients were referred elsewhere because they could not be managed in the hospital, primarily due to a lack of space for administration. Considering that patients referred to another hospital could undergo hip fracture surgeries, the combined operational definition’s actual sensitivity will increase when applied to the national claims database, which includes information from all medical institutes in South Korea. Second, an operational definition was created only for OHFs and no other osteoporotic fractures (i.e., vertebral, wrist, and proximal humerus) [27].

Nevertheless, this proposed approach can guide future validation studies on the operational definitions for each type of fracture. Based on the current literature, this is the first study to verify the operational definition of hip fractures using administrative data on older adults in South Korea. This study presents the possibility of developing operational definitions for other treatment areas using diagnosis codes and procedure codes in claims data. As the use of real-world administrative data increases, the methodology used in developing an algorithm for an operational definition of osteoporotic fracture in this study can be applied for similar purposes in studies on other diseases in the future. In order to discriminate true OHFs and reduce the risk of incorrectly including false OHFs, orthopedic surgeons independently reviewed various medical records from a hospital equipped with a highly accurate electronic medical record system.

## 5. Conclusions

The developed operational definition of OHFs based on a combination of diagnosis code levels and procedure codes for hip fracture surgeries is proved to be a valid tool for identifying patients with OHFs in the claims database. Moreover, the overall accuracy and discriminative power of correctly identifying patients with OHFs increased as more levels of diagnosis codes and appropriate procedure codes were used in health insurance claims data. This study’s findings may be of great value and importance to health policy makers when developing guidelines to prevent and treat OHFs, as experiencing OHFs deteriorates quality of life and reduces life expectancy significantly while also increasing the burden of treatment costs. Further studies are recommended to validate the operational definition’s accuracy using big data sets.

## Figures and Tables

**Table 1:** Patients’ characteristics.

Characteristic	Total (N = 522) N (%)	Female (N = 344) N (%)	Male (N = 178) N (%)	*p*-Value
Age (mean ± SD)	71.14 (±11.74)	73.07 (±11.49)	67.40 (±11.34)	<0.0001
50–59	104 (19.9)	49 (14.2)	55 (30.9)	<0.0001 *
60–74	208 (39.9)	136 (39.5)	72 (40.5)	
≥75	210 (40.2)	159 (46.2)	51 (28.7)	
N of patients with hip fractures based on:				
Primary Dx codes				
S72.0 or S72.1	134 (25.7)	99 (28.8)	35 (19.7)	0.0238
S72.0	65 (12.5)	47 (13.7)	18 (10.1)	0.2442
S72.1	69 (13.2)	52 (15.1)	17 (9.6)	0.0751
Primary or secondary Dx codes				
S72.0 or S72.1	136 (26.1)	101 (29.4)	35 (19.7)	0.0167
S72.0	65 (12.5)	47 (13.7)	18 (10.1)	0.2442
S72.1	71 (13.6)	54 (15.7)	17 (9.6)	0.0521
Primary, secondary, or tertiary Dx codes				
S72.0 or S72.1	172 (32.9)	125 (36.4)	47 (26.4)	0.0221
S72.0	85 (16.3)	61 (17.7)	24 (13.5)	0.2126
S72.1	88 (16.9)	65 (18.9)	23 (12.9)	0.0839

*p*-value, *t*-test for continuous variables and chi-square test for count data; N, number; SD, standard deviation; Dx, diagnosis. * *p*-value for all age groups using chi-square test.

**Table 2:** Patients’ diagnosis codes according to the diagnosis code levels listed in the claims data.

Dx Codes	Primary Dx Code	Primary or Secondary Dx Codes	Primary, Secondary, or Tertiary Dx Codes	Primary vs. Primary, Secondary, or Tertiary
	(N = 522) N (%)	(N = 522) N (%)	(N = 522) N (%)	*p*-Value
S72.0 (Fracture of neck of femur)	65 (12.5)	65 (12.5)	85 (16.3)	<0.0001
S72.1 (Pertrochanteric fracture)	69 (13.2)	71 (13.6)	88 (16.9)	<0.0001
M87.0 (Idiopathic aseptic necrosis of bone)	95 (18.2)	95 (18.2)	99 (19.0)	0.0455
M16.9 (Coxarthrosis, unspecified)	120 (23.0)	120 (23.0)	120 (23.0)	1.0000
T84.0 (Mechanical complication of an internal joint prosthesis)	22 (4.2)	22 (4.2)	23 (4.4)	0.3173
M00.9 (Pyogenic arthritis, unspecified)	13 (2.5)	13 (2.5)	13 (2.5)	1.0000
Z47.0 (Persons encountering health services for follow-up care involving removal of fracture plate and other internal fixation devices)	8 (1.5)	8 (1.5)	9 (1.7)	0.3173

N, number; Dx, diagnosis; *p*-value, McNemar’s chi-square test for count data of primary codes vs. primary, secondary, or tertiary codes.

**Table 3:** Association between algorithms using Dx with PRO codes and orthopedic surgeons in identifying hip fractures.

Algorithms Using Dx with PRO Codes from Claims Data	Hip Fractures Identified from Claims Data	Hip Fractures Confirmed by Orthopedic Surgeons through Medical Record Review			
		Yes (N = 134)		No (N = 388)	
		N	(%)	N	(%)
Based on primary Dx codes only					
Dx codes only	Yes	131	(97.8)	29	(7.5)
	No	3	(2.2)	359	(92.5)
Dx and PRO codes	Yes	131	(97.8)	29	(7.5)
	No	3	(2.2)	359	(92.5)
Dx or PRO codes	Yes	158	(35.8)	2	(2.5)
	No	283	(64.2)	79	(97.5)
Based on primary and secondary DX codes					
Dx codes only	Yes	132	(97.1)	28	(7.3)
	No	4	(2.9)	358	(92.8)
Dx and PRO codes	Yes	132	(97.8)	28	(7.2)
	No	3	(2.2)	359	(92.8)
Dx or PRO codes	Yes	158	(35.8)	2	(2.5)
	No	284	(64.3)	78	(97.5)
Based on primary, secondary, and tertiary Dx codes					
Dx codes only	Yes	155	(90.1)	5	(1.4)
	No	17	(9.9)	345	(98.6)
Dx and PRO codes	Yes	154	(95.7)	6	(1.7)
	No	7	(4.4)	355	(98.3)
Dx or PRO codes	Yes	159	(35.2)	1	(1.4)
	No	293	(64.8)	69	(98.6)

N, number; Dx, diagnosis; PRO, procedure.

**Table 4:** Validity of the operational definition for osteoporotic hip fractures.

Identification Using:	Sensitivity	Specificity	Positive Predictive Value	Negative Predictive Value	Accuracy	AU-ROC Curve
	% (95% CI)	% (95% CI)	% (95% CI)	% (95% CI)	% (95% CI)	% (95% CI)
Primary Dx code						
Dx code only	81.9 (75.0–87.5)	99.2 (97.6–99.8)	97.8 (93.6–99.5)	92.5 (89.4–94.9)	93.9 (91.5–95.8)	0.91 (0.88–0.94)
Dx and PRO codes	81.9 (75.0–87.5)	99.2 (97.6–99.8)	97.8 (93.6–99.5)	92.5 (89.4–94.9)	93.9 (91.5–95.8)	0.91 (0.88–0.94)
Dx or PRO codes	98.8 (95.6–99.8)	21.8 (17.7–26.4)	35.8 (31.3–40.5)	97.5 (91.4–99.7)	45.4 (41.1–49.8)	0.60 (0.58–0.63)
Primary and secondary Dx codes						
Dx codes only	82.5 (75.7–88.0)	98.9 (97.2–99.7)	97.1 (92.6–99.2)	92.7 (89.7–95.1)	93.9 (91.5–95.8)	0.91 (0.88–0.94)
Dx and PRO codes	82.5 (75.7–88.0)	99.2 (97.6–99.8)	97.8 (93.6–99.5)	92.8 (89.7–95.1)	94.1 (91.7–95.9)	0.91 (0.88–0.94)
Dx or PRO codes	98.8 (95.6–99.8)	21.5 (17.4–26.1)	35.7 (31.3–40.4)	97.5 (91.3–99.7)	45.2 (40.9–49.6)	0.60 (0.58–0.62)
Primary, secondary, and tertiary Dx codes						
Dx codes only	96.9 (92.9–99.0)	95.3 (92.6–97.2)	90.1 (84.6–94.1)	98.6 (96.7–99.5)	95.8 (93.7–97.3)	0.96 (0.94–0.98)
Dx and PRO codes	96.3 (92.0–98.6)	98.1 (96.1–99.2)	95.7 (91.2–98.2)	98.3 (96.4–99.4)	97.5 (95.8–98.7)	0.97 (0.96–0.90)
Dx or PRO codes	99.4 (96.6–100.0)	19.1 (15.1–23.5)	35.2 (30.8–39.8)	98.6 (92.3–100.0)	43.7 (39.4–48.1)	0.59 (0.57–0.61)

AU-ROC curve, area under the receiver operating characteristic curve; 95% CI, 95% confidence interval; Dx, diagnosis; PRO, procedure.

## Data Availability

Not applicable.
